# Blood Flow after Endovascular Repair in the Aortic Arch: A Computational Analysis

**DOI:** 10.1055/s-0039-1683771

**Published:** 2019-02-22

**Authors:** Theodorus M. van Bakel, Rodrigo M. Romarowski, Simone Morganti, Joost A. van Herwaarden, Frans L. Moll, Hector W. de Beaufort, Massimiliano M. Marrocco-Trischitta, Francesco Secchi, Michele Conti, Ferdinando Auricchio, Santi Trimarchi

**Affiliations:** 1 Thoracic Aortic Research Center, IRCCS—Policlinico San Donato, University of Milan, Milan, Italy; 2 Department of Vascular Surgery, University Medical Center Utrecht, Utrecht, The Netherlands; 3 Department of Civil Engineering and Architecture, University of Pavia, Pavia, Italy; 4 Department of Radiology, IRCCS—Policlinico San Donato, San Donato Milanese, Italy; 5 Department of Biomedical Sciences for Health, University of Milan, Milan, Italy

**Keywords:** thoracic endovascular aortic repair, blood flow, displacement force

## Abstract

**Background** The benefits of thoracic endovascular aortic repair (TEVAR) have encouraged stent graft deployment more proximally in the aortic arch. This study quantifies the hemodynamic impact of TEVAR in proximal landing zone 2 on the thoracic aorta and the proximal supra-aortic branches.

**Methods** Patients treated with TEVAR in proximal landing zone 2 having available preoperative and 30-day postoperative computer tomography angiography and phase-contrast magnetic resonance imaging data were retrospectively selected. Blood flow was studied using patient-specific computational fluid dynamics simulations.

**Results** Four patients were included. Following TEVAR in proximal landing zone 2, the mean flow in the left common carotid artery (LCCA) increased almost threefold, from 0.21 (0.12–0.41) L/min to 0.61 (0.24–1.08) L/min (+294%). The surface area of the LCCA had not yet increased commensurately and therefore maximum flow velocity in the LCCA increased from 44.9 (27.0–89.3) cm/s to 72.6 (40.8–135.0) cm/s (+62%). One of the patients presented with Type Ib endoleak at 1-year follow-up. The displacement force in this patient measured 32.1 N and was directed dorsocranial, perpendicular to the distal sealing zone. There was a linear correlation between the surface area of the stent graft and the resulting displacement force ( *p* = 0.04).

**Conclusion** TEVAR in proximal landing zone 2 alters blood flow in the supra-aortic branches, resulting in increased flow with high flow velocities in the LCCA. High displacement forces were calculated and related to stent graft migration and Type I endoleak during 1-year follow-up.

## Introduction

Thoracic endovascular aortic repair (TEVAR) has decreased perioperative mortality and morbidity in treatment of diseases of the descending thoracic aorta. 1 2 The benefits of endovascular treatment have encouraged most vascular surgeons to deploy stent grafts also more proximally into the aortic arch. Despite the less invasive character of TEVAR in the aortic arch, stroke remains an important complication associated with significant morbidity and mortality. 3

Early on in the adoption of TEVAR as first-line treatment, a classification for preoperative planning of TEVAR in the aortic arch was proposed, identifying four proximal landing zones in the ascending aorta and aortic arch: zone 0 to 3. 4 Stent graft deployment in zones 0, 1, and 2 determines the coverage of one or more proximal supra-aortic branches, which is generally managed by creating an extra-anatomical bypass, redirecting blood flow.

TEVAR in zone 2 is frequently accompanied by a left carotid-to-subclavian bypass and is performed in one-third or more of patients undergoing TEVAR. 5 The benefits of embolization or revascularization of the left subclavian artery (LSA) are still subject of debate. 6 7

The hemodynamic effects of TEVAR in zone 2 are not well understood. Zone 2 deployment is generally considered safe. 7 However, there is an increased risk of stroke 8 and Type I endoleak 9 compared with deployment in the descending thoracic aorta, distally to the origin of the LSA (LSA). Calculation of the hemodynamic effects of stent graft deployment in zone 2 may provide additional information to help understand the pathophysiology of these complications. In this setting, computational fluid dynamics (CFD) simulations can be performed to calculate the blood flow in patient-specific models of the aorta. The aim of this study was to use this powerful tool to quantify the impact of TEVAR in zone 2 on blood flow in the aorta and supra-aortic branches.

## Methods

### Patient Selection

The database of IRCCS Policlinico San Donato was retrospectively quarried for patients who underwent zone 2 TEVAR for thoracic aortic diseases between 2013 and 2016. Patients who had available preoperative and 30-day postoperative computer tomography angiography (CTA) and phase-contrast magnetic resonance imaging (PC-MRI) data were included. Clinical data at 30-day and 1-year follow-up were noted. This study was approved by the local ethics committee, which waived the need for patients' informed consent due to the retrospective nature of the analysis and the use of anonymized data.

### Medical Imaging

The standard CTA protocol for aortic imaging at our institution includes a 64-multislice computed tomography (CT) scan (SOMATOM Definition AS, Siemens, Germany) after administration of a contrast agent (Iomeron 370, Bracco, Milan, Italy). Image reconstructions were done with 1 mm slice thickness. CTA imaging data were used for measurement of the surface areas of the proximal supra-aortic branches and constructing the models for CFD simulations.

The protocol for PC-MRI in patients with aortic pathologies at our institution uses a 1.5 Tesla MRI scanner (MAGNETOM Aera, Siemens, Erlangen, Germany). Flow measurements were taken at the level of the ascending aorta, brachiocephalic trunk (BCT), left common carotid artery (LCCA), LSA, and descending aorta, and were used to set the inflow and outflow boundary conditions for the CFD simulations.

### Computational Fluid Dynamics Simulations

Three-dimensional models of the thoracic aorta were constructed from CTA imaging data using the Vascular Modeling Toolkit level set segmentation (VMTK software suite version 1.3). The models included the ascending aorta, aortic arch, proximal part of the supra-aortic branches, and descending thoracic aorta. Once the models were segmented, a computational mesh was created to follow the fluid domain. The surface was smoothed with a Taubin algorithm to avoid shrinking.

CFD simulations were performed to retrieve blood flow velocity, pressure, and wall shear stress fields using previously described techniques. 10 Boundary conditions were imposed in a patient-specific manner. In particular, flow profiles as extracted from each patient's pre- and postoperative PC-MRI were imposed at the ascending aorta. On the outflow sections, classic three-element Windkessel circuits were applied to mimic the compliance and resistance of the distal vascular bed. 11 12 The Windkessel models were calibrated to match the patient-specific blood pressure, from brachial artery cuff measurement, and flow measurements from PC-MRI. Convergence of velocity and pressure data are a requirement to obtain meaningful CFD simulation results. 12 Therefore, CFD simulations of six cardiac cycles were performed, and results from the last cardiac cycle were used. Post-processing was done using open source software ParaView version 4.1.0 (Kitware Inc, Los Alamos National Laboratory, NM).

### Outcome Measures

CFD simulations were performed using preoperative and 30-day postoperative imaging data. Mean flow was calculated in the ascending aorta, BCT, LCCA, LSA, and descending aorta. Blood flow through the ascending aorta, proximal supra-aortic branches, and descending aorta was calculated before and after intervention.

Flow velocity measurements were taken just distal to the origin of the proximal supra-aortic branches on the center lumen line.

Hemodynamic displacement forces acting on the surface of the stent graft were calculated from postoperative CFD simulation results ( Eq. 1 ). 13 14



The displacement force (DF) in Newton is calculated by the sum of the pressure ( *p* ) and wall shear stress ( *τ* ) on the surface area of the stent graft ( *dA* ) in systolic peak.

### Statistical Analysis

Data were analyzed using SPSS statistics version 24 (IBM, Armonk, NY). Continuous data are reported as mean values with the range given between brackets. Differences between the pre- and postoperative results are reported in percentages. Correlations were calculated using the Pearson's correlation coefficient. Statistical significance was assumed at *p* < 0.05.

## Results

### Patient Characteristics

Four patients were selected ( Table 1 ). All patients were treated with TEVAR in zone 2 using Medtronic Valiant stent grafts (Medtronic Vascular, Santa Rosa, CA) and were submitted to a left carotid-to-subclavian bypass within the same procedure.

**Table 1:** Patient characteristics

Patient	Age (y)	Sex	Disease	Stent graft dimensions (mm)
1	48	M	TAA	28–24–150
2	65	M	PAU	42–42–100
3	74	M	TAA	Combined 44–44–200/46–46–200/46–46–150
4	81	M	TAA	Combined 42–42–200/46–46–200/46–46–150

Abbreviations: M, male; PAU, penetrating aortic ulcer; TAA, thoracic aortic aneurysm.

Stent graft dimensions are presented in the following order: proximal diameter–distal diameter–length.

At the 30-day postoperative visit, clinical follow-up was uneventful for all patients. At 1-year follow-up, patient 4 presented with a Type Ib endoleak. Patients 1, 2, and 3 were free of complications.

### Mean Flows

**Fig. 1:**
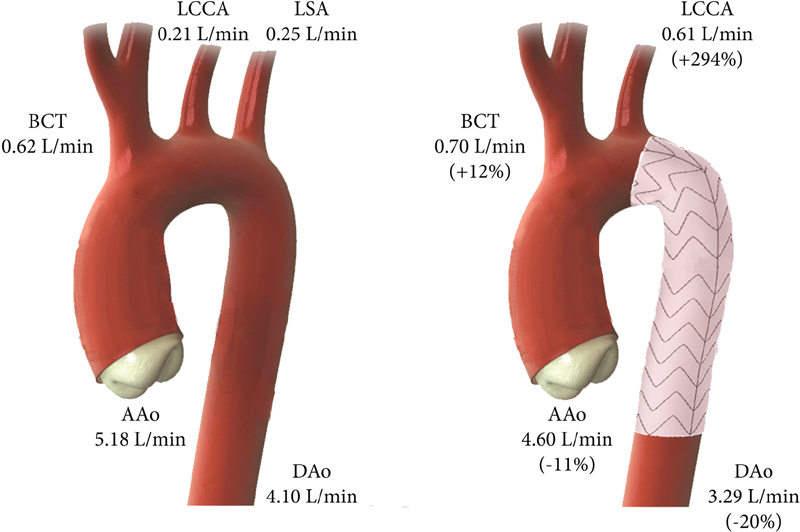
Blood flow following TEVAR in zone 2. Mean flows in the ascending aorta, proximal supra-aortic branches, and descending aorta before TEVAR in zone 2 and after 30-day follow-up. The difference is given within brackets. AAo, ascending aorta; BCT, brachiocephalic trunk; DAo, descending aorta; LCCA, left common carotid artery; LSA, left subclavian artery.

Fig. 1 shows the mean flows before TEVAR and after 30-day follow-up; data are given in Table 2 . Before TEVAR, the mean flow in the ascending aorta was 5.18 (3.69–6.31) L/min. Mean flow in the BCT was 0.62 (0.54–0.75) L/min, in the LCCA 0.21 (0.12–0.41) L/min, in the LSA 0.25 (0.17–0.33) L/min, and in the descending aorta 4.10 (2.25–5.26) L/min.

**Table 2:** Blood flow before and after TEVAR in zone 2

Patient	Before TEVAR			LSA	DAo	After TEVAR			
	AAo	BCT	LCCA			AAo	BCT	LCCA	DAo
1	6.31	0.54	0.19	0.33	5.26	6.64	1.21	1.08	4.35
2	5.98	0.67	0.12	0.17	5.01	4.61	0.57	0.24	3.81
3	4.74	0.54	0.12	0.24	3.85	4.53	0.72	0.76	3.05
4	3.69	0.75	0.41	0.28	2.26	2.62	0.30	0.37	1.95
Mean	5.18	0.62	0.21	0.25	4.10	4.60	0.70	0.61	3.29

Abbreviations: AAo, ascending aorta; BCT, brachiocephalic trunk; DAo, descending aorta; LCCA, left common carotid artery; LSA, left subclavian artery; TEVAR, thoracic endovascular aortic repair.

Blood flow measurements are presented in L/min.

Following TEVAR, the mean flow in the LCCA increased almost threefold (+294%), whereas blood flow in the ascending aorta and descending aorta decreased (−11% and −20%, respectively).

### Surface Areas of Proximal Supra-aortic Branches

The surface areas of the proximal supra-aortic branches were measured on preoperative and 30-days postoperative CT images. Data are given in Table 3 . Before TEVAR, the surface area of the BCT was 3.16 (0.95–6.09) cm ^2^ , the surface area of the LCCA was 0.94 (0.39–1.44) cm ^2^ , and the surface area of the LSA was 1.61 (0.75–2.9) cm ^2^ . At 30-day follow-up, the surface area of the BCT did not change (+1%). The surface area of the LCCA increased by 9%.

**Table 3:** Surface area of the proximal supra-aortic branches before and after TEVAR in zone 2

Patient	Before TEVAR		LSA	After TEVAR	
	BCT	LCCA		BCT	LCCA
1	0.95	0.39	0.75	1.05	0.57
2	2.22	0.67	0.82	2.10	0.76
3	3.40	1.27	1.95	3.53	1.30
4	6.09	1.44	2.90	6.06	1.46
Mean	3.16	0.94	1.61	3.19	1.02

Abbreviations: BCT, brachiocephalic trunk; LCCA, left common carotid artery; LSA, left subclavian artery; TEVAR, thoracic endovascular aortic repair.

Surface areas were measured just distal to the origin from the aortic arch and are given in cm ^2^ .

### Flow Velocities

Data of maximum flow velocity in the proximal supra-aortic branches are given in Table 4 . Before TEVAR, maximum flow velocity in the BCT was 47.9 (14.1–104.4) cm/s, in the LCCA 44.9 (27.0–89.3) cm/s, and in the LSA 47.6 (13.3–105.3) cm/s.

**Table 4:** Maximum flow velocity in the proximal supra-aortic branches

Patient	Before TEVAR		LSA	After TEVAR	
	BCT	LCCA		BCT	LCCA
1	104.4	89.3	105.3	106.7	134.9
2	36.6	27.0	25.8	33.7	40.8
3	35.9	33.8	45.9	28.3	71.4
4	14.1	29.3	13.3	8.9	43.2
Mean	47.8	44.9	47.6	44.5	72.6

Abbreviations: BCT, brachiocephalic trunk; LCCA, left common carotid artery; LSA, left subclavian artery; TEVAR, thoracic endovascular aortic repair.

Maximum flow velocity before TEVAR in zone 2 and after 30-day follow-up was measured just distal to the origin from the aortic arch and given in cm/s.

**Fig. 2:**
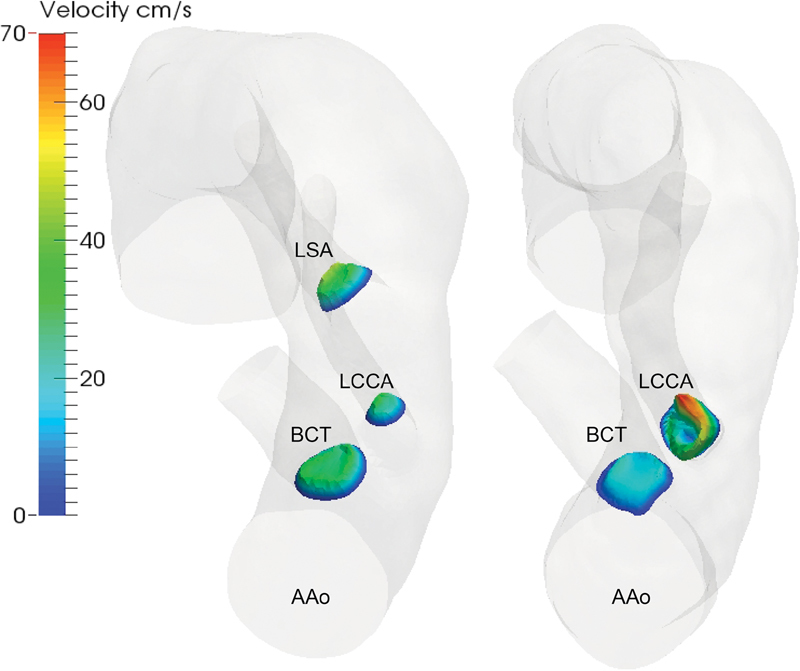
Velocity contours in the proximal supra-aortic branches. Peak flow velocity is mapped just distal to the origin of the proximal supra-aortic branches. AAo, ascending aorta; BCT, brachiocephalic trunk; LCCA, left common carotid artery; LSA, left subclavian artery.

Following TEVAR, maximum flow velocity in the LCCA increased by +62%. Fig. 2 presents a mapping of the peak systolic flow profiles in the proximal supra-aortic branches of patient 3 before and after TEVAR.

### Displacement Forces

**Fig. 3:**
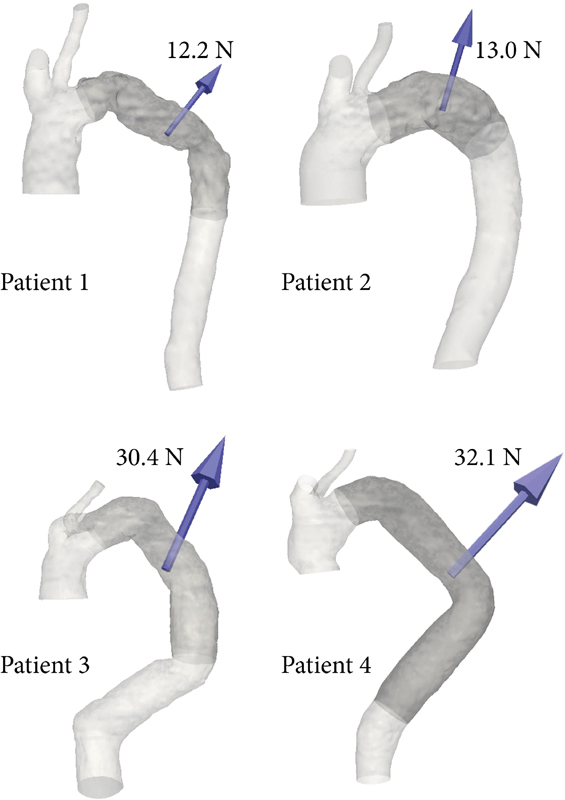
Displacement forces acting on the stent grafts. The displacement force vectors are shown in all four patients. The contours of the stent grafts are outlined in dark gray. The magnitude of the displacement force is given in Newton.

The magnitude of the displacement force acting on the stent grafts ranged from 12.2 to 32.1 N. The displacement force vector was directed dorsocranial in all patients ( Fig. 3 ).

**Fig. 4:**
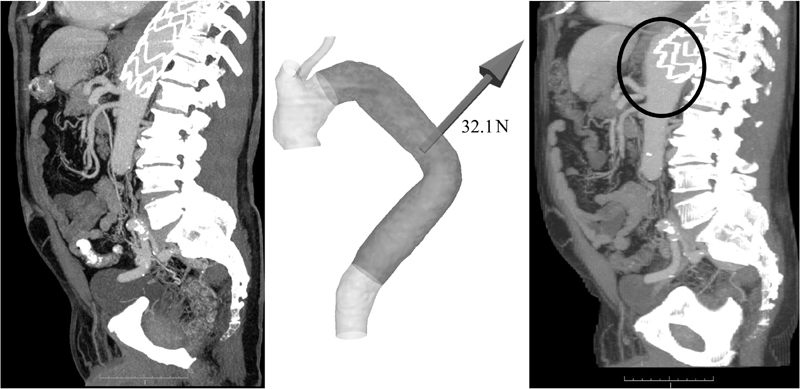
Displacement force and Type Ib endoleak in patient 4. On the left, a sagittal CT image of the postoperative situation is shown. In the middle, the postoperative model with the calculated displacement force vector is presented. On the right, a sagittal CT image at 1-year follow-up is shown, where proximal migration of the stent graft and resulting Type Ib endoleak was found (marked with circle).

Patient 4 presented with Type Ib endoleak at 1-year follow-up. The center of the distal end of the stent graft migrated 17.1 mm dorsocranial in the direction of the displacement force ( Fig. 4 ).

**Fig. 5:**
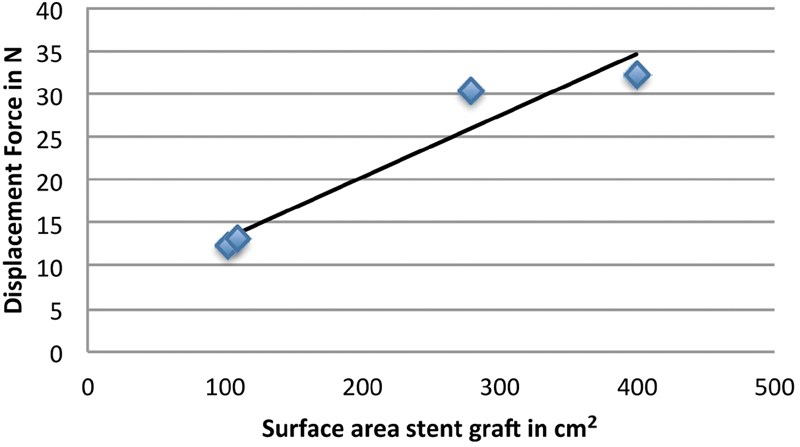
Stent graft surface area and resulting displacement force. The surface areas of the stent grafts are plotted against the peak systolic displacement forces. A trend line is added visualizing the linear correlation.

The magnitude of the displacement force had a linear correlation with the surface area of the stent graft ( *p* = 0.04), ( Fig. 5 ). Although the total surface area of the stent graft in patient 3 was not as large as in patient 4, the magnitude of the displacement force was similar. This is caused by the higher blood pressure in patient 3 compared with patient 4. Blood pressure measurements from CFD simulation results are reported in Table 5 .

**Table 5:** Blood pressure measurements from CFD simulation results

Patient	Before TEVAR		Mean	After TEVAR		Mean
	Systolic	Diastolic		Systolic	Diastolic	
1	131	58	82	127	66	87
2	110	49	80	108	56	80
3	117	61	89	114	65	88
4	94	62	77	106	55	77
Mean	113	58	82	114	61	83

Abbreviations: CFD, computational fluid dynamics; TEVAR, thoracic endovascular aortic repair.

Blood pressures measurements were taken from CFD simulation results at the ascending aorta and are given in mm Hg.

## Discussion

In this study, we used patient-specific CFD simulations to quantify some of the hemodynamic effects of TEVAR in zone 2 on blood flow in the aorta and the supra-aortic branches. Our results show that blood flow predominantly changed in the proximal LCCA, where flow increased almost threefold. The vessel size of the LCCA did not change commensurately at 30-day follow-up, and consequently flow velocity increased.

Little is known about the effects of TEVAR in zone 2 on blood flow in the carotid arteries. Our results show that blocking the LSA and redirecting blood flow to the left arm via the LCCA and left carotid-to-subclavian bypass increases flow velocities in the proximal LCCA. This is likely to induce arterial remodeling in the proximal LCCA during follow-up. 15 The correlation between flow alterations and intimal thickening and stenosis has been investigated extensively. 16 Moreover, increased flow velocities induce elevated endothelial shear stresses, which are not only a triggering factor for arterial remodeling but also atherosclerotic plaque formation and inflammatory processes. 17 18 Besides inducing arterial remodeling, the outflow tract of the left carotid-to-subclavian bypass can be a relatively low-resistance path compared with the distal LCCA, especially if a stenosis is present distal to the bypass, resulting in subclavian steal from the LCCA. Further research is warranted to study these scenarios and the impact of different treatment options, such as the use of a branched stent graft versus left carotid-to-subclavian bypass, on blood flow in the carotid and vertebral arteries following zone 2 TEVAR.

Previous studies showed the relationship between displacement forces and stent graft migration in the abdominal aorta. 19 Compared with the abdominal aorta, the displacement force is significantly larger and directed cranial in the thoracic aorta. 14 20 Stent graft size is a key determinant of the magnitude of the displacement force. 21 Our results confirm these findings. The patient in whom we had calculated the highest displacement force, measuring 31.1 N, presented with Type Ib endoleak after 1-year follow-up. The displacement force calculated in this patient lies within the range of pullout forces described by Rahmani et al. 22 Interestingly, we calculated a similar displacement force in patient 3, measuring 30.4 N; however, no migration or endoleak was found in this case. We hypothesize that the direction of the displacement force being perpendicular to the distal sealing zone might have attributed to the stent graft being pulled out of its original position.

Further investigation of the hemodynamic displacement force and the complex of compensatory radial and frictional forces, securing stability of the stent graft in the sealing zones, is required. In the future, these mechanisms could potentially be taken into account in preoperative planning for TEVAR, thus reducing the risk of Type I endoleak.

### Limitations

A limitation of the CFD simulations performed for this study is the rigid wall assumption. More accurate simulations would include fluid structure interaction, for which computational costs are significantly higher. 23

We simulated only flow through arteries for which we had patient-specific flow measurements; therefore, we did not include the coronary and distal carotid arteries in our models.

Our CFD simulation results showed increased flow velocities in the LCCA. Future prospective studies that focus on the impact of TEVAR in zone 2 on cerebral blood flow should also include flow measurements more distal in the internal and external carotid arteries and in the vertebral arteries.

The small number of patients included in this study limits the impact of the results. Although clear trends were found, studies consisting of a larger study population are required to confirm our findings.

## Conclusion

TEVAR in proximal landing zone 2 alters blood flow in the supra-aortic branches, resulting in increased flow with high flow velocities in the LCCA. Our results warrant further investigation of cerebral blood flow following TEVAR in the aortic arch. The use of large stent grafts in the thoracic aorta results in high displacement forces, increasing the risk of stent graft migration and Type I endoleak during follow-up.
